# Intraluminal Migration of a Penrose Drain Presented with Hematochezia, after Lower Gastrointestinal Surgery

**DOI:** 10.1055/s-0042-1757603

**Published:** 2022-10-10

**Authors:** Roza Mourelatou, Christos Liatsos, Angeliki Bistaraki, Efstathios Nikou

**Affiliations:** 1 2nd Department of General Surgery, 417 Army Share Fund Hospital, Athens, Greece; 2 Department of Gastroenterology, 401 Army General Hospital, Athens, Greece; 3 Department of Nursing, School of Health Sciences, Hellenic Mediterranean University, Athens, Greece; 4 2nd Department of General Surgery, 401 Army General Hospital, Athens, Greece

**Keywords:** drain migration, penrose, intraluminally, lower GI surgery, hematochezia

## Abstract

**Background** Although surgical drains are widely used after lower gastrointestinal (GI) procedures, complications may occur. Specifically, sporadic cases of drain migration into a hollow viscus, most commonly regarding active drains and treated with surgical removal, have been reported. Herein, we present a case of a passive drain (penrose) migration into the colon, after segmental sigmoidectomy with primary anastomosis, presented with hematochezia.

**Methods** A 37-year-old male patient suffering from colovesical fistula, due to sigmoid diverticulitis, underwent resection of the fistula, the involved sigmoid segment and the bladder opening, followed by primary anastomosis of the colon and primary closure of the bladder. A penrose catheter was positioned near the anastomosis.

**Results** On 8th postoperative day (POD) the patient had three episodes of hematochezia and blood in the drain collection bag, followed by relative improvement. On 15th POD gas was observed on the drain's collection bag and a new episode of hematochezia led him to sigmoidoscopy. The endoscopy revealed the presence of the penrose drain intraluminally, protruding via an ulcer at the level of the anastomosis. The penrose repositioned outside the lumen and metallic clips were used to approximate the defect. The patient was then fully recovered, discharged, and the drain removed on follow-up.

**Conclusion** To our knowledge this is the first report of drain migration presented with hematochezia, after lower GI surgery, avoided reoperation, and resolved with removal of the drain under direct endoscopic vision.

Surgical drains are commonly used prophylactically to prevent fluid accumulation or therapeutically after surgical procedures and are classified as either active (low or high negative pressure closed drain system) or passive (penrose). Surgical drain-related complications, such as fragmentation, pain, infection, loss of function due to obstruction, and perforation of visceral organs, have been described, more commonly with vacuum drains. 1 Drain complications were related to prolonged hospitalization, invasive intervention for nonfunctional/removed drains, and also emergency surgery in 8 of 2,004 patients (0.4%) and an important contributing factor found to be the psychological status of the patient, as previously reported. 2

Sporadic cases had emerged in bibliography, reporting migration of variety of medical objects into hollow viscera: sponges, 3 4 hernia meshes, 5 and jejunostomy tubes. 6 7 On the other hand, antegrade migration of a jejunostomy tube has been described by Prahlow and Barnard. Their study mentioned the peristalsis induced intraluminal antegrade migration of the tube's distal end with concomitant retrograde movement of the small bowel over the tube. 8 Furthermore, the translocation of a foreign object into a hollow viscera has also been reported, regarding an adjustable gastric band migration into the stomach, after bariatric surgery in 3.1% of the cases. 9

Other complications of drain placement, including infection, pain, herniation, perforation, hemorrhage, and irritation to the surrounding tissues, have been previously reported. In the same study the author mentioned fracture of drains, which most commonly occurred at the level of the suture that retains the drain to the abdominal wall and also described a technique to prevent this complication. 10 On the same perspective fragmentation and migration in the surgical field had been observed in other foreign objects, such as hernia mesh, due to inadequate stabilization. 11 12

Only a few cases worldwide have reported intraluminal migration of surgical drains and much less reported perforation of the bowel. The aim of our study was to present a case of intraluminal migration of a penrose drain into the sigmoid colon, after a segmental sigmoidectomy for the treatment of colovesical fistula, which presented with episodes of hematochezia.

## Materials and Methods

A 37-year-old male Caucasian patient admitted to our department for elective treatment of colovesical fistula, due to complicated diverticulitis. No other findings emerged from his medical history. Presurgical lower gastrointestinal (GI) endoscopy revealed at 20 cm of the anus, an area of mucosal edema and minor redness, of 10 cm of length, with diverticular orifices. The contrast radiographic studies showed leakage of the contrast media inserted from the anus, to the bladder.

The patient underwent laparotomy with resection of the fistula and the involved segment of the sigmoid colon with primary anastomosis, rejuvenation of the fistula orifice, and primary closure of the bladder. After vigorous hemostasis, a penrose drain was inserted in the peritoneal cavity and placed near the anastomosis. The patient tolerated well the general anesthesia, extubated, and moved to the recovery unit.

## Results

Patient's recovery was uneventful until the 4th postoperative day (POD), when he presented with fever, with elevated inflammation markers, white blood cells, and C-reactive protein, but with normal dismissal of gases and feces. On the 8th POD he presented three episodes of hematochezia, with no abdominal pain, with further elevation of inflammation markers and reduction of hematocrit, without the need for transfusion. The fluid discharge via the penrose drain had 50 mL of blood clot that day, despite the previous normal yellowish appearance. The patient was then started on total parenteral nutrition, stopped per os feeding, and received upgraded antibiotics. The next days he did not exhibit any signs of sepsis and also had gradual clinical and laboratory improvement.

**Fig. 1:**
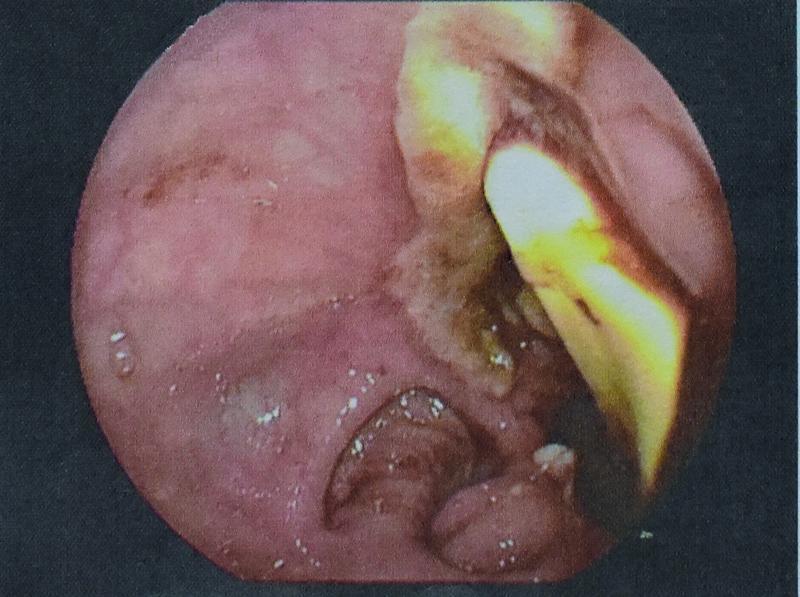
Endoscopical caption of penrose drain protruding intraluminally, from ulcer in sigmoid anastomosis.

**Fig. 2:**
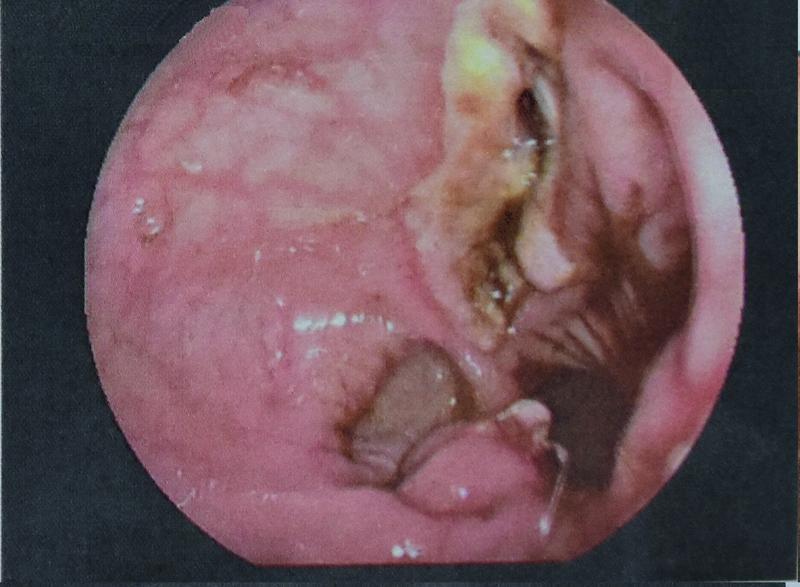
Endoscopical caption of the ulcer in sigmoid anastomosis, after reposition of the drain into the peritoneal cavity.

**Fig. 3:**
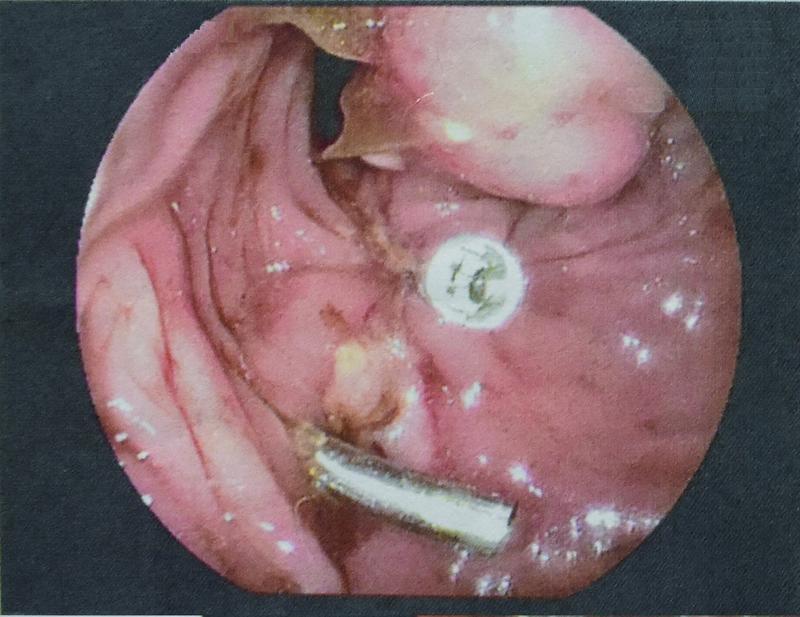
Clip placement to approximate the defect, endoscopically.

On the 15th POD gas was observed on the collection bag of the drain, followed by a new episode of hematochezia, accompanied by 300 mL bloody fluid discharge via the drain. After stabilization of the patient with intravenous fluids and transfusion with blood products, a sigmoidoscopy was decided. In the sigmoid colon at the level of the anastomosis, an ulcer was identified. On the base of the ulcer the penrose drain was protruding intraluminally ( Fig. 1 ). The penrose repositioned outside the bowel lumen ( Fig. 2 ), into the peritoneal cavity and metallic clips were used to approximate the defect ( Fig. 3 ).

Afterwards the patient's course was improved and discharged a few days later with the drain in place. During follow-up on 30th POD, the patient underwent a new sigmoidoscopy, which revealed the known ulcer, which exhibited signs of normal healing process and the drain was finally removed.

## Discussion

Reviewing the literature regarding the appearance of foreign bodies in hollow viscus and specifically surgical drains, most of the cases of drain migration were observed after upper GI surgery. On 2007 Wilmot et al reported the intraluminal migration of a Jackson-Pratt catheter into the region of the anastomosis in 1.6% of the patients who underwent transhiatal esophagogastrectomy, equal to 7% of the patients who developed an anastomotic leak, as a postsurgical complication. The study highlighted the need of drain withdrawal or removal to facilitate healing of the anastomotic leak. 13

In 2010 Lai et al referred to intraluminal migration of vacuum drain 13 mm into the esophagojejunostomy, after total gastrectomy and proceeded with removal of the drain under direct endoscopic observance. Afterwards constant suction of the nasogastric tube for 5 days allowed the ulcer to heal. 14 The same incident was observed with a Jackson-Pratt drain protruding through the gastrojejunal anastomosis after gastric bypass. In that case a leak was first diagnosed with upper GI series, followed by endoscopy due to failure of clinical improvement, which finally put the diagnosis and allowed direct vision during the removal of the drain. 15 It is of note that in all the above cases reoperation was avoided.

In compliance with the aforementioned incidents we decided to proceed with endoscopy, which enabled the diagnosis and also the treatment, because repositioning of the penrose drain into the peritoneal cavity and insertion of metallic clips in the ulcer's base was crucial to initiate the healing process of the ulcer and the clinical improvement of the patient.

The etiology of this phenomenon still remains unclear, but in the case of vacuum/aspiration drains Nomura et al proposed that the drain may draw the bowel wall, initiating a necrotic process leading to perforation of the bowel wall, and finally penetration of the drain into the lumen. 16 The underlining mechanism leading to intraluminal migration of a penrose, that is, a passive drain, is yet uncertain, due to the rarity of the cases.

Regarding the diagnosis of the incidence, in several cases of intraluminal migration of a drain, after upper GI surgery was made with upper GI radiographic series. 13 In other cases, as in our patient, the diagnosis was confirmed during endoscopy, which also enabled the treatment. 14 15 On the other hand, Subhash et al reported intraluminal position of a Foley catheter and the diagnosis made intraoperatively, 17 same with the case of Micalef et al, which presented with sepsis and signs of bowel perforation and the migration of the drain was found 3 months later during exploratory laparotomy for ileostomy closure. 18

The responsible object in the cases of migration of a surgical drain with bowel perforation was a vacuum drainage, 19 a silicone drain, 16 and a Foley catheter. 17 Intestinal intraluminal migration of foreign bodies, especially surgical drains constitutes an uncommon complication after colectomy. Migration of a Foley, used as drain, into the transverse colon after segmental resection of the transverse colon, with complete insertion of the catheter into the peritoneal cavity and extraction of the drain was possible during reoperation, has been previously described. 17

Regarding the type of drain, to our knowledge, only two reports mentioned intraluminal migration of a penrose catheter into the ileum 18 and a penrose fragment into the small bowel, months after surgery for obstructed incisional hernia after anterior resection for sigmoid diverticulitis. In this case computed tomography revealed a foreign body inside the small bowel, which needed surgical removal. 20

To our knowledge this is the first report of drain migration presented with GI bleeding, after lower GI surgery, avoided reoperation, and resolved minimally invasively, with endoscopic removal of the drain and closure of the defect using clips.
